# Discriminative ability of adiposity measures for elevated blood pressure among adolescents in a resource-constrained setting in northeast Nigeria: a cross-sectional analysis

**DOI:** 10.1186/s40608-018-0211-7

**Published:** 2018-12-03

**Authors:** Oghenebrume Wariri, Iliya Jalo, Fidelia Bode-Thomas

**Affiliations:** 1 Vaccines and Immunity Theme, MRC Unit, The Gambia at The London School of Hygience and Tropical Medicine, Fajara, The Gambia; 2 Department of Paediatrics, Federal Teaching Hospital (FTH), Gombe, Nigeria; 3 Department of Paediatrics, College of Medical Sciences, Gombe State University, Gombe, Nigeria; 4 Paediatric Cardiology Unit, Department of Paediatrics, Jos University Teaching Hospital (JUTH), Jos, Nigeria

**Keywords:** Measures of adiposity, Body mass index, Waist circumference, Waist-to-height ratio, Prehypertension, Hypertension, Elevated blood pressure, Nigeria

## Abstract

**Background:**

Several studies examining the association and discriminative ability of adiposity measures for prehypertension and hypertension among adolescents have reported varying outcomes. We aimed to determine the discriminative ability of the Body Mass index (BMI), Waist Circumference (WC), and Waist-to-Height Ratio (WHtR) adiposity measures for elevated blood pressure (prehypertension and hypertension combined) among adolescents in Gombe, northeast Nigeria.

**Methods:**

This cross-sectional study used a multi-stage sampling technique and involved 367 secondary school adolescent (10–18 years) boys and girls in Gombe Local Government Area, Gombe State, northeast Nigeria from January to September 2015. We examined and compared the associations and discriminative ability of the BMI, WC and the WHtR for elevated blood pressure using multiple logistic regression and receiver operating characteristics (ROC) curves. Area under the curves (AUC), odds ratio (OR) and 95% confidence intervals (CI) are reported.

**Results:**

All three measures of adiposity were strongly and positively associated with elevated blood pressure. The BMI obesity showed the strongest association with elevated blood pressure with odds that was double the odds of WC and triple that of WHtR [adjusted OR for BMI 15.3, 95% CI (4.8–27.9)]. The discriminative ability of adiposity measures for elevated blood pressure using AUC was comparable (0.786 for BMI, vs 0.780 for WC, vs 0.761 for WHtR).

**Conclusion:**

We provide evidence, here on the BMI, WC and WHtR to support the use of simple indirect measures of adiposity in evaluating adiposity-related risk including prehypertension and hypertension among Nigerian adolescents.

**Electronic supplementary material:**

The online version of this article (10.1186/s40608-018-0211-7) contains supplementary material, which is available to authorized users.

## Background

Childhood overweight and obesity currently pose major public health challenges, with associated increase in adiposity-related health risk including hypertension [1]. Although Africa accounts for a significant proportion of the total number of undernourished children globally, the number of overweight and obese children in the continent increased steeply from 5.4 million in 1990 to 10.3 million children in 2015 [2–4]. The post-2015 Sustainable Development Goals have identified obesity as a core priority for prevention and control if the world must reach the goal on achieving health for all by 2030 [1].

Empirical evidence from longitudinal studies shows that a significant proportion of primary hypertension is associated with the epidemic of overweight and obesity, even in the adolescent age group [5, 6]. For example, there is almost a 10 fold risk of elevated blood pressure among BMI classified overweight or obese adolescents compared to normal weight categories [7].

Epidemiological studies evaluating the relationship between excess adiposity and the development of hypertension in adolescents have traditionally used the measures of general, or truncal adiposity. However, the BMI, a measure of general adiposity is considered not discriminatory of the distribution of body fat in the abdominal region, which is now considered the critical factor in the development of obesity-related hypertension [8, 9]. Measures of truncal adiposity such as the WC and WHtR, compared to the BMI are currently being considered as better predictors of adiposity-related health risk, including hypertension, in populations of all ages based on evidence from predominantly Caucasian literature [10, 11].

Several empirical studies [12–15] which compared the BMI, WC and WHtR reported that the WHtR, has significantly higher discriminatory ability for, or is more predictive of the associated risk of hypertension compared to the WC and BMI. This is thought to be because truncal adiposity measured by the WHtR adjusts for height while accounting for visceral adiposity which is currently considered the critical factor in the development of cardiovascular disease including hypertension [13, 15]. A drawback of these empirical studies however, is that most of them involved exclusively Caucasian populations, making it to difficult to extrapolate their findings to predominantly African populations with different genetic and environmental factors that determine body fat patterning. Some earlier studies in Nigerian children [16–18], and adolescents [19, 20] however, linked large BMI and WC to an increased risk of elevated blood pressure in children but with different outcomes in terms of their discriminative abilities.

There is limited literature on the relationship between obesity that is determined by the WHtR and risk of elevated blood pressure in Nigerian children and adolescents. The WHtR, is a measure of truncal adiposity that is relatively easy to obtain, does not require the use of reference tables to determine categories (unlike the WC or BMI), and uses a single cut-off value of 0.5 to define obesity in both adults and children [21–23]. Our aim, therefore, was to determine which measure of adiposity (BMI, WC, and WHtR) was most discriminative of elevated blood pressure among adolescents in northeast Nigeria.

## Methods

### Study setting and population

We conducted a cross-sectional analytical study involving apparently healthy secondary school adolescents in Gombe Local Government Area (LGA) of Gombe State between January 2015 and September 2015. Gombe State which is mainly populated by the Fulani, Hausa, Bolewa, Waja, Tera and Tangale ethnic groups is located in the northeast of Nigeria and shares borders with Yobe, Borno, Adamawa, Bauchi, and Taraba States [24].

Gombe LGA which also includes Gombe town, the capital of the State is the largest among 11 other LGAs and has a population of 302,674 with children aged 18 years and below being approximately 50% of the population [24].

**Fig. 1:**
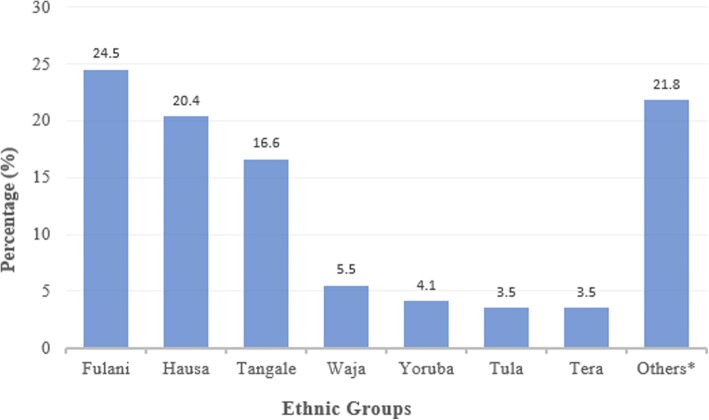
Bar chart showing distribution of Ethnic nationalities of 367 study participants in percentages. Others* (Babur, Bagazime, Berom, Bolewa, Bura, Cham, Dadiya, Ebira, Igbo, Jukun, Kanuri, Kare-Kare, Kushi, Lunguda, Margi, Ngamo, Tiv, Wurkum)

Among study participants, five ethnic groups accounted for more than 70% of study participants: Fulani 90 (24.5%), Hausa 75 (20.4%), Tangale 61 (16.6%), Waja 20 (5.5%), and Yoruba 15 (4.1%). However, there were 25 ethnic nationalities among the study participants (Fig. 1).

### Sampling

A multistage random sampling technique was used in this study to recruit 377 adolescents aged 10–18 years from 12 secondary schools including six public and six private schools respectively in Gombe LGA. The number recruited was based on an estimation that used a prevalence of hypertension of 5.4% from a previous Nigerian study [25], a power of 80%, degree of precision of 2.5%, a 95% confidence interval and allowing for 20% loss due to incomplete data. In the initial stage, six (6) out of the eleven (11) political Wards in Gombe LGA were selected by simple balloting to increase representativeness. In each of the selected Wards, the list of all the secondary schools (based on information from the Gombe State ministry of education) was made out by ownership, i.e., public and private schools. One public and one private school were selected from each Ward by simple balloting as well. Thus, a total of 12 schools were selected from the six Wards (i.e., two schools per ward, one private and one public). The number of students recruited from each school, and subsequently from each grade was determined by the proportionate sampling method.

After determining the number of participants to be recruited from each grade, the class register of each grade (the sampling frame) was used and the participants were selected using a systematic sampling technique. The sampling interval was determined by dividing the total number of students in each grade by the number of participants selected from that grade. A number was randomly selected from a list of random numbers and was considered the initial point for sampling. Participants who did not fit the inclusion criteria were omitted and the next student on the list was subsequently selected.

### Inclusion and exclusion criteria

Secondary school adolescents aged 10–18 years from the selected schools whose parents/guardians had consented to their participation in the study and who themselves have assented to the study were included. Participants excluded from the study include; those with any form of chronic disease based on participant volunteered information, available school records, or evidence from physical examination. Other exclusion criteria were presence of haematuria and glucosuria on urinalysis, participants who actively consumed alcohol or cigarette within the past 3 months to the date of the study and participants who were on any mediction known to affect blood pressure such as steroids, and diuretics.

### Data collection

Pre-tested questionnaires (Additional file 1) were administered to parents/guardians to fill-out, and was subsequently completed by participants, individually on the day of study. The questionnaire contained information on participants’ sociodemographic characteristics, drug history, parental education/occupation and family history of hypertension. Anthropometric parameters and blood pressure measurements were taken and recorded in the school premises by the researchers and trained assistants as described below.

### Anthropometry and blood pressure measurement

All participants removed their outer clothing, accessories, shoes, belts, wrist watches and emptied their pockets before measurements were taken. Body weight was measured to the nearest 0.1 kg using a digital scale (Seca® 877 Class III). Height was measured to the nearest 0.1 cm using a potable, collapsible stadiometer (Seca® Leicester portable height measure). Waist circumference were measured according to standard procedures with a non-stretch tape rule (Seca® 2011) placed horizontally, once, midway between the lower border of the 10th rib and the top of the iliac crest, at normal expiration [26].

Blood pressure measurements were done per the recommendations of the 4th report criteria of the National High Blood Pressure Education Programme [5]. Measurements were taken at the level of the heart with participants in seated position, using a standard mercury sphygmomanometer (ACCUSON® Hospital model BS 274) with systolic and diastolic blood pressure read off at the 1st and 5th Korotkoff respectively. Systolic and diastolic blood pressures were calculated as the mean of three readings taken 1 week apart.

### Derived variables

BMI was derived from the formula: BMI = weight (kg)/height (m)^2^.

WHtR was derived using the formula: WHtR = waist circumference (cm)/height (cm) [27].

### Operational definition of variables

1. Prehypertension was any blood pressure ≥90th percentile but < the 95th percentile or any blood pressure >120/180 mmHg [5].
2. Hypertension was any blood pressure ≥95th percentile [5].
3. Elevated blood pressure was any blood pressure ≥90th percentile (for clinical relevance, i.e. prehypertension and hypertension inclusive) [5].
  Adult cut-off values of blood pressure ≥140/90 mmHg for hypertension, systolic blod pressure = 120–139 mmHg, and diastolic blood pressure = 80–89 mmHg for prehypertension, were used for participants, 18 years old [28].
4. Overweight was defined as:
  - WC ≥ 75th percentile but <90th percentile [29].
  - BMI ≥ 85th percentile but <97th percentile [30].
5. Obesity was defined as:
  - WC ≥90th percentile [29].
  - BMI ≥97th percentile [30].
  - WHtR ≥0.5 [27].

### Data analysis

All data generated were processed and analysed using the IBM Corp SPSS statistics for windows version 24.0 (Armonk, NY: IBM Corp). To determine which measures of adiposity (the independent variables) was strongly associated with or discriminative of elevated blood pressure (the dependent variable), multiple logistic regression models were fitted. The regression models were adjusted for known confounders of blood pressure in children such as age, gender, height, parental socioeconomic status and school type of participants.

The adjusted odds ratio (AOR), and 95% CI (i.e., the odds of ‘elevated blood pressure’ in one category of adiposity compared to participants with normal adiposity within same measure of adiposity) were subsequently reported and compared to the odds using other measures of adiposity.

Receiver operating characteristics (ROC) curves and associated area under the curves (AUCs) were generated for all three measures of adiposity to further estimate the discriminatory ability for elevated blood pressure. Furthermore, the overall discriminatory ability for elevated blood pressure was determined by combining all three measures of adiposity.

The AUC is a measure of overall discriminatory ability of each adiposity measure, with a value of 0.5 representing no discriminative ability and 1.0 representing perfect discrimination of elevated blood pressure. The AUCs were compared using a non-parametric approach for comparison of multiple AUCs from ROC curves [31].

## Results

### Demographics, adiposity measures, and blood pressures

A total of 377 adolescents were recruited to participate in this study. Of these, 370 participants who fulfilled the study criteria eventually completed the study. Data for 367 participants were analysed, because three participants were excluded due to incomplete or missing data at the time of data analysis. There were 191 (52%) boys and 176 (48%) girls, making a boys to girls ratio of 1.1:1, with the mean age of study participants being 14.9 ± 1.9 years. There was no significant difference in the mean age of boys and girls (*p* = 0.780).

The mean weight of study participants was 49.6 ± 12.2 kg. Girls in comparison to boys of same age categories were generally heavier, and the differences in weight was statistically significant with *p* = 0.001 (Tables 1 and 2). The mean WC was 68.7 ± 9.6 cm. Girls compared to boys of the same age categories generally had larger WC, the differences were statistically significant early and middle adolescence (Table 2).

**Table 1:** Background characteristics of study participants by categories of BMI, WC and WHtR (*N* = 367)

Variable	BMI Categories				WC Categories				WHtR Categories		
	Normal	Overweight	Obese	*P*	Normal	Overweight	Obese	*P*	Normal	Obese	*P*
	*n* (%)				*n* (%)				*n* (%)		
Participants gender											
Boys	176(57.5)	6(16.2)	9(37.5)		179(54.6)	7(25.9)	5(41.7)		179(57.2)	12(22.2)	
Girls	130(42.5)	31(83.8)	15(62.5)	0.001*	149(45.4)	20(74.1)	7(58.3)	0.013*	134(42.8)	42(77.8)	0.001*
Adolescence age group											
Early	68(22.2)	9(24.3)	12(50.0)		70(21.3)	12(44.5)	7(58.3)		70(22.4)	19(35.2)	
Middle	154(50.3)	16(43.2)	10(41.7)		165(50.3)	11(40.7)	4(33.3)		159(50.8)	21(38.9)	
Late	84(27.5)	12(32.4)	2(8.3)	0.024*	93(28.4)	4(14.8)	1(8.4)	0.003*	84(26.8)	14(25.9)	***0.107***
Parental socioeconomic status^a^											
Low	96(31.4)	5(13.5)	5(20.8)		97(29.6)	7(25.9)	2(16.6)		97(31.0)	9(16.7)	
Medium	131(42.8)	14(37.8)	7(29.2)		144(43.9)	5(18.6)	3(25.0)		134(42.8)	18(33.3)	
High	79(25.9)	18(48.6)	12(50.0)	0.001*	87(26.5)	15(55.5)	7(58.4)	0.001*	82(26.2)	27(50.0)	0.009*
School type											
Public	173(56.5)	15(40.5)	5(20.8)		183(55.8)	8(29.6)	2(16.7)		174(55.6)	19(35.2)	
Private	133(43.5)	22(59.5)	19(79.2)	0.001*	145(44.2)	19(70.4)	10(83.3)	0.001*	139(44.4)	35(64.8)	0.010*

***n*** **(%)** = Frequency (percentage)

^a^Classified by combining scores from maternal education and paternal occupation as described by Olusanya et al. [46]

*Significant *p*-values

**Table 2:** Distribution of adiposity measures and blood pressures of study participants (*N* = 367)

Variable	Boys	Girls	*p*-value	Total	
	*N* = 191	*N* = 176		*N* = 367	*p*-value
Weight (kg)					
Early adolescence	41.8 (14.1)	48.0 (12.1)	*0.030*	44.8 (13.5)	
Middle adolescence	46.9 (10.8)	52.4 (11.5)	*0.001*	49.6 (11.5)	
Late Adolescence	54.3 (8.9)	53.9 (12.1)	*0.860*	54.1 (10.4)	0.001*
Body Mass index					
Early adolescence	18.7 (4.9)	20.6 (4.4)	*0.053*	19.6 (4.7)	
Middle adolescence	18.27 (2.8)	21.2 (4.6)	*0.001*	19.7 (4.1)	
Late Adolescence	19.4 (4.7)	21.4 (4.1)	*0.005*	20.3 (3.7)	0.393*
Waist circumference (cm)					
Early adolescence	66.2 (12.8)	72.1 (9.8)	*0.018*	69.1 (11.8)	
Middle adolescence	65.3 (7.1)	70.9 (9.8)	*0.001*	68.1 (8.9)	
Late Adolescence	69.3 (6.5)	70.0 (10.7)	*0.713*	69.6 (8.6)	0.415*
Waist-to-height ratio					
Early adolescence	0.44 (0.07)	0.47 (0.06)	*0.046*	0.46 (0.07)	
Middle adolescence	0.41 (0.03)	0.45 (0.06)	*0.001*	0.43 (0.05)	
Late Adolescence	0.41 (0.04)	0.44 (0.06)	*0.009*	0.42 (0.05)	0.001*
Blood pressure categories *n* (%)					
Prehypertensive	19 (9.1)	20 (11.4)	0.787**	39 (10.6)	
Hypertensive	8 (4.2)	13 (7.4)	0.274**	21 (5.7)	

Values presented as mean (SD), unless otherwise indicated, ***n*** **(%)** = Frequency (percentage), *ANOVA *p*-values reported, **Chi-square *p*-values reported. Early adolescence (10–13 years), Middle adolescence (14–16 years), Late adolescence (17–18 years) as described by Cromber et al. [47]

The mean BMI of study participants was 19.8 ± 4.1 kg/m^2^ and girls in comparison to boys of the same age group categories generally had larger, and the differences were statistically significant during middle and late adolescence (Table 2). The mean WHtR was 0.44 ± 0.06, with girls compared to the boys having larger WHtR across all age group categories. The gender differences in WHtR was statistically significant across all adolescence categories.

The overall prevalence of prehypertension was 10.6% while that of hypertension was 5.7% with girls having higher prevalence compared to boys in our study, however, these differences were not statistically significant (Table 2).

### Adiposity measures and predictive/discriminative ability for elevated blood pressure

**Fig. 2:**
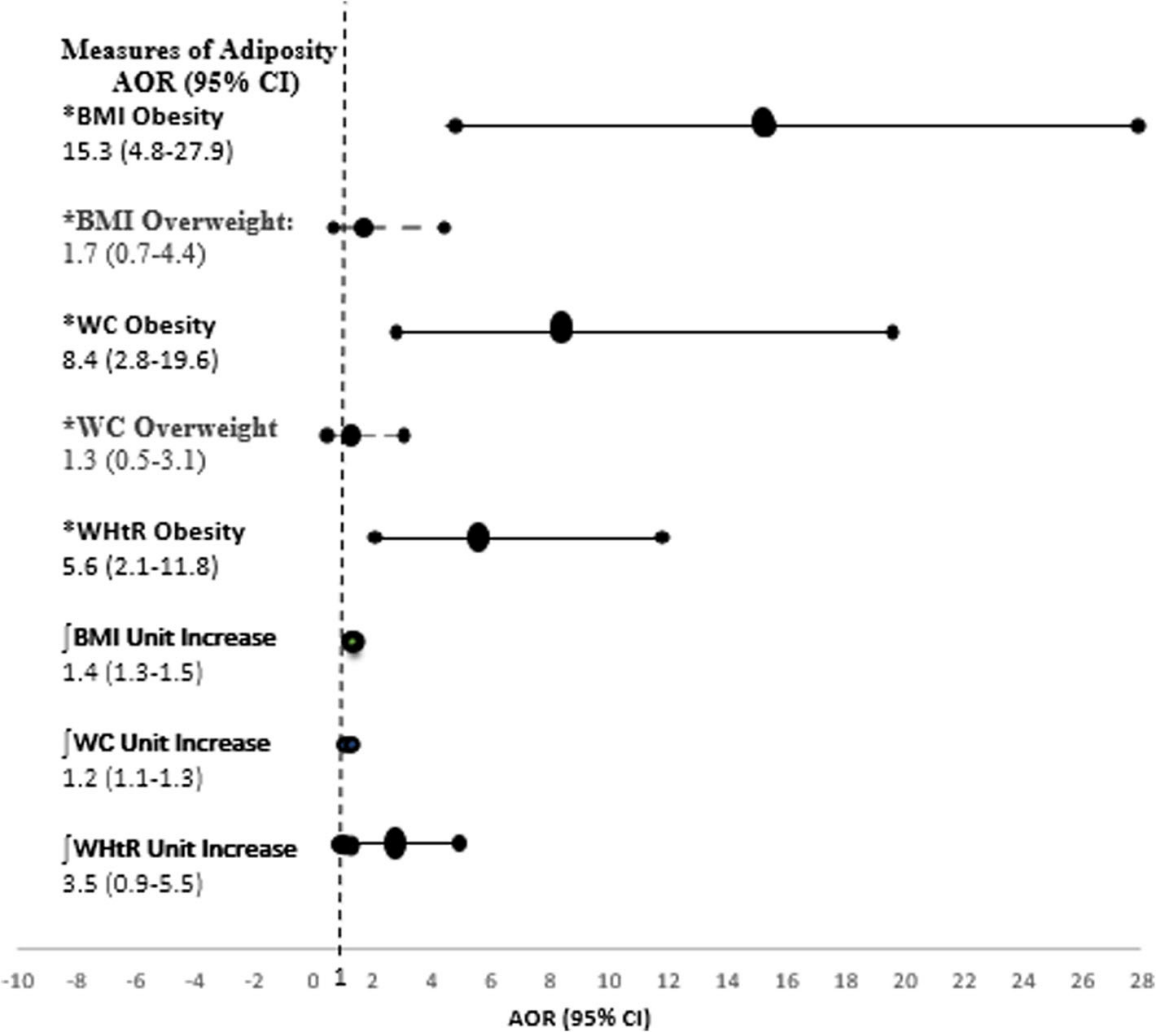
Comparative odds ratios for **Elevated Blood Pressure** by categories and unit increase of adiposity measure among adolescents (*N* = 367). **BMI and WHtR** OR was adjusted for parental socioeconomic status, school type, age, and sex, while **WC** was adjusted for parental socioeconomic status, school type, height, age and gender. **Elevated blood pressure** was categorized by combining prehypertension and hypertension. *Represent AOR of discriminatory ability of elevated blood pressure by BMI, WC, and WHtR adiposity categories. ∫Represent AOR of discriminatory ability of elevated blood pressure per unit increase in centimetre for BMI, WC and WHtR

The comparative AOR and confidence intervals for the predicted odds of elevated blood pressure by measures of adiposity are shown in the forest plot in Fig. 2, while secondary analysis showing the predicted odds of prehypertension and hypertension separately is shown in Table 3. Overweight measured by the BMI compared to the WC, showed the strongest association with elevated blood pressure. Obesity measured by the BMI compared to the WC and WHtR also showed the strongest association with elevated blood pressure [AOR = 15.3 (95% CI = 4.8–27.9), *p* < 0.001], followed by the WC and WHtR respectively (Fig. 2).

**Table 3:** Secondary analysis of predcited odds of preypertnsion and hypertension by categories and unit increase of adiposity measures among participants (*N* = 367)

Variable	Prehypertension		Hypertension	
	AOR (95% CI)	*p*-value	AOR (95% CI)	*p*-value
Body Mass Index				
Obesity	7.45 (2.34, 13.75)	*0.001*	25.22 (9.52, 46.81)	*0.001*
Overweight	1.18 (0.35, 3.84)	*0.109*	4.90 (3.30, 8.91)	*0.001*
^a^Unit increase	0.88 (0.75, 1.04)	*0.110*	1.60 (1.20, 1.70)	*0.023*
Waist circumference				
Obesity	3.42 (0.74, 8.82)	*0.070*	11.08 (7.77, 24.28)	*0.001*
Overweight	1.25 (0.27, 4.43)	*0.450*	2.65 (0.93, 7.57)	*0.090*
^a^Unit increase	0.99 (0.92–1.07)	*0.570*	1.40 (1.22, 1.52)	*0*.*045*
Waist-to-Height Ratio				
Obesity	2.53 (0.64, 0.87)	*0.530*	11.18 (5.36, 23.29)	*0*.*001*
^a^Unit increase	3.33 (0.96, 11.59)	*0.054*	5.42 (1.25, 8.48)	*0.020*

^a^Represent AOR of discriminatory ability of elevated blood pressure per unit increase in centimetre for BMI, WC and WHtR

**Fig. 3:**
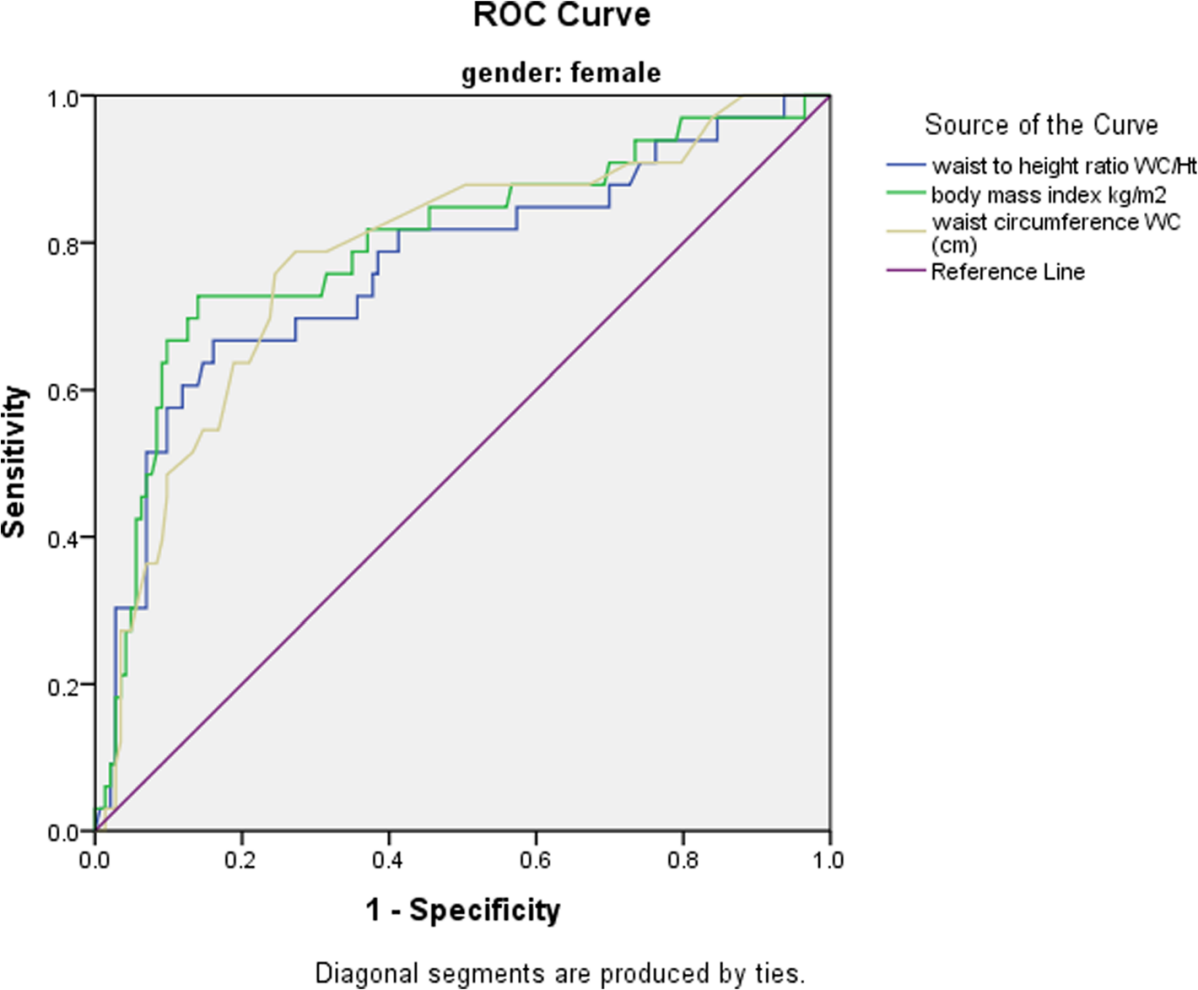
Receiver operating characteristics curve for discriminative ability of the BMI, WC, and WHtR adiposity measures for elevated blood pressure

**Fig. 4:**
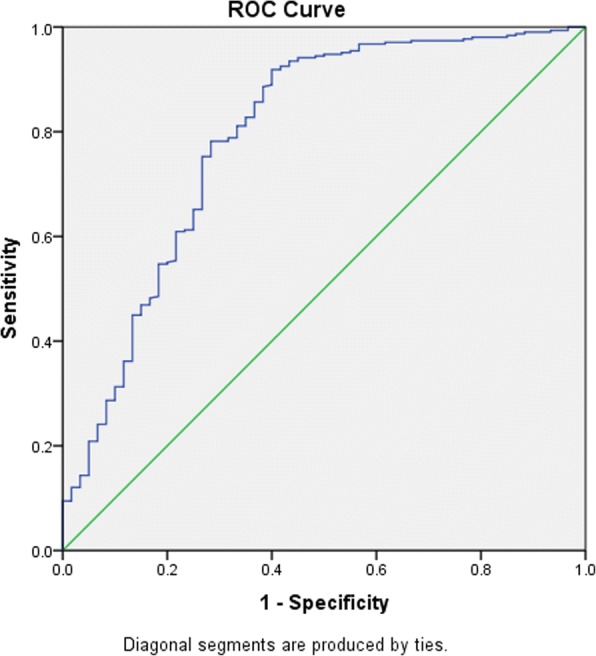
Receiver operating characteristics curve for discriminative ability for elevated blood pressure by combining the three adiposity measures (i.e. BMI, WC andWHtR Combined)

Shown in Figs. 3 and 4 are the ROC curves of the discrimination of elevated blood pressure by each measures of adiposity, and when all three measures were combined respectively, while Table 4 shows the AUCs by gender. Regardless of gender, all adiposity measures demonstrated significant discriminatory ability for elevated blood pressure with AUC ranging from 0.75 to 0.80. The BMI was however the most discriminatory, followed by WC and WHtR in that order, while the discriminatory ability improved overall when the measures were combined, compared to using a single measure. Girls had higher discriminatory ability for elevated blood pressure compared to boys using the same adiposity measure. Nevertheless, the relative gender-specific differences for AUC between measures of adiposity was negligible (Table 4).

**Table 4:** Area under the curve for discriminative ability of elevated blood pressure by adiposity measures among participants (*N* = 367)

Variable	Boys	Girls	Overall
	AUC^a^ (95% CI)	AUC^a^ (95% CI)	AUC^a^ (95% CI)
Body Mass Index	0.770 (0.67, 0.87)	0.790 (0.70, 0.90)	0.786 (0.71, 0.86)
Waist circumference	0.760 (0.66, 0.88)	0.780 (0.70, 0.87)	0.780 (0.70, 0.85)
Waist-to-Height Ratio	0.750 (0.64, 0.87)	0.770 (0.67, 0.87)	0.761 (0.69, 0.84)
Combined Measures	0.779 (0.67, 0.89)	0.798 (0.71, 0.90)	0.800 (0.74, 0.86)

^a^*AUC* Area Under the Curve

## Discussion

The major strength of our study was that we compared the discriminatory ability of three measures of adiposity; BMI WC, and WHtR for elevated blood pressure among adolescents in a resource-constained setting of northeast Nigeria. The BMI, a measure of general adiposity compared to the WC and WHtR which are measures of truncal adiposity had the strongest association and discriminatory ability for elevated blood pressure. The odds of elevated blood pressure using the BMI was double the odds using the WC and triple the odds of elevated blood pressure using the WHtR respectively. Our finding is comparable to two similar studies done in South-western Nigeria which reported that the BMI was the most discriminatory or had the highest strength of association with elevated blood pressure among adolescents comparatively to measures of truncal obesity [20, 32].

However, our findings are in contrast to several empirical studies, systematic reviews, and meta-analyses [12–15, 33, 34] among non-African or predominantly Caucasian environments which reported that the measures of truncal adiposity, especially the WHtR were the most predictive, or discriminatory of cardiovascular risk including elevated blood pressure even among adolescents. These studies argued that the size of truncal fat deposit is better correlated with visceral adipose tissue volume which is considered a major risk factor for cardiovascular risk, including elevated blood pressure compared to measures general adiposity.

This contrast in evidence may be explained by the fact that differential adipose tissue or fat patterning is race and ethnicity specific. Caucasians are generally more likely to deposit adipose tissue centrally in the trunk while in Africans it is more likely around the hips [35]. This scenario may support the increase in the discriminative ability of measures of truncal adiposity compared to measures of general adiposity for elevated blood pressure among Caucasians.

Additionally, the available cut-off points used to classify WC and WHtR truncal obesity are derived based on data from predominantly Caucasian or non-African populations. The cut-off points for WC overweight and obesity for example, may be smaller for Nigerian adolescents and even more so in our study setting due to chronic undernutrition among children and adolescents in the northeast which accounts for a major proportion of wasting and stunting in Nigeria [36]. Therefore, using the available cut-offs to interpret measures of Nigerian, and more specifically northeast Nigerian adolescents with different pattern of body fat deposition, genetic make-up, and chronic undernutrition may not yield similar results. Thus the WC and WHtR, may not show similar discriminatory ability or association with cardiovascular risk such as elevated blood pressure like those reported in the predominantly Caucasian literature.

The BMI is the most widely used adiposity measure to determine obesity worldwide [37]. The WHO chart and cut-off points which is used to classify adiposity in this study was developed based on longitudinal data from a WHO study conducted across six countries worldwide, including Ghana, with similar genetic and environmental factors which determine body fat distribution like Nigeria [30]. The fact that the chart was developed based on data from Caucasian and African populations, ensured that important environmental and genetic factors specific to the these populations were considered in determining the optimal cut-offs. Thus, may be more discriminatory of elevated blood pressure in a Nigerian population like in our study and other Nigerian studies [20, 32], compared to the measures of truncal adiposity which were derived based on data from predominantly Caucasian population.

The measures of adiposity were more discriminative for elevated blood pressure among girls with consistenty higher AUC across the three adiposity measures compared to boys. The significantly higher proportion of overweight and obese adiposity categories among girls may have accounted for the higher discriminative ability for elevated blood pressure in girls using these measures. Hormonal changes such as increased steroid hormone secretion which determine body fat deposition and blood pressure occur a bit earlier in girls than in boys of the same age during puberty [38]. These changes may result in more girls being overweignt and obese earlier, with higher predicted odds of elevated blood pressure as shown in our data. Our finding is similar to another Nigerian study [39] which also found a significantly higher proportion of overweight and obese girls with corresponding increased predicted odds of elevated blood pressure by the BMI and WC among girls.

Given the strong positive association and comparability of AUCs for the discriminative ability of adiposity measures for elevated blood pressure in our study (0.79 for BMI, vs 0.78 for WC, vs 0.76 for WHtR respectively), the use of WC and WHtR may however also be sufficient in clinical settings for determining associated elevated blood pressure. The WHtR as a measure of truncal adiposity for example is an easy to use [40], non-invasive measure of truncal adiposity [41], correlates well with visceral fat [42], and uses a single cut-off in determing obesity in children and adults [43]. These characteristic makes the WHtR specifically adaptable in resource-constrained clinical settings of sub-Saharan Africa that lack trained paediatric or other health workforce needed for implementing and interpreting the BMI which is comparatively more complex.

Although the WHtR is easy to use in clinical and population settings, does not require complex calculations and reference to age, gender, and race-specific tables, data on WHtR is still limited in Nigerian adolescents [43]. Further studies among Nigerian adolescents to determine appropriate cut-off points for classifying WC or WHtR adiposity are however required to aid interpretation of its discriminatory ability for elevated blood pressure in African populations.

There are limitations to our study. Firstly, due to the cross-sectional nature of our study, causal and temporal inferences between the measures of adiposity and elevated blood pressure may not be made for the associations reported. However, the largely causal relationship between excess adiposity and elevated blood pressure is well-established [44, 45], and the reverse causality is implausible. Secondly, the WHtR cut-off of 0.5 which is a cardiovascular risk predictor classifies abdominal adiposity into normal and obsese categories only, compared to the BMI and WC which have overweight and obese categories separately. Therefore, the difference between normal and obese categories classified by the BMI, WC and WHtR may be slightly different and may impact the interpretation of our findings. Lastly, the generalizability of our findings to other settings may be limited by the fact that our study was a school-based study.

## Conclusion

We provide evidence, here on the BMI, WC and WHtR to support the use of simple measures of adiposity in evaluating adiposity-related risk among Nigerian adolescents. The BMI, a measure of general adiposity showed the strongest association or discriminatory ability for the presence of elevated blood pressure compared to the measures of truncal adiposity. Although the WC and WHtR showed lesser discriminatory ability for elevated blood pressure, they may still have important clinical implication for risk-stratification in adolescent population in resource limited settings where determination of more complex adiposity measures like the BMI may not be feasible.

## Additional file

Additional file 1: Study questionnaire developed specifically for use in this study. (DOCX 16 kb)

## Abbreviations

AUC: Area Under the Curve
BMI: Body Mass Index
CI: Confidence Interval
LGA: Local Government Area
OR: Odds Ratio
ROC: Receiver Operating Characteristics
SD: Standard Deviation
SPSS: Statistical Package for the Social Sciences
WC: Waist Circumference
WHO: World Health Organization
WHtR: Waist-to-Height Ratio
