# Supplementary material for: Discriminative ability of adiposity measures for elevated blood pressure among adolescents in a resource-constrained setting in northeast Nigeria: a cross-sectional analysis
Source: BMC Obes. 2018 Dec 3;5:35. doi: 10.1186/s40608-018-0211-7 (PMC6276203; doi:10.1186/s40608-018-0211-7)
Supplement: Supplementary file 1 — Study questionnaire developed specifically for use in this study. (DOCX 16 kb) [file 40608_2018_211_MOESM1_ESM.docx]

**Additional file 1:** Study Questionnaire.

**RESEARCH TOPIC:** MEASURES OF ADIPOSITY AND RISK OF ELEVATED BLOOD PRESSURE IN APPARENTLY HEALTHY SCHOOL ADOLESCENTS IN GOMBE LOCAL GOVERNMENT AREA, GOMBE STATE, NORTHEAST NIGERIA.

***Please fill or tick as appropriate***  **Pupil’s code no: ____**

**Name of school___________________________ Date ___________**

**Class _______**

(A) PUPIL’S BIODATA (*to be filled by PARENT or GUARDIAN*)

(A_1_) **Age at last birthday_________**

(A_2_) **Gender** Male **[ ]** Female **[ ]**

(A_3_) **Religion** Islam [ ] Christianity [ ] Traditional [ ] Others [ ]

(A_4_) **Tribe ________**

(B) FAMILY/PARENTS’ DATA (*to be filled by PARENT or GUARDIAN*)

(B_1_) **Highest education of Parent or Guardian completed: (**tick as appropriate)

**Father Mother**

No formal education [ ] [ ]

Primary education [ ] [ ]

Secondary [ ] [ ]

Tertiary education;

*Below university (NCE/OND)* [ ] [ ]

*Up to university (HND, BSc, PhD)* [ ] [ ]

Others (Specify) __________________ I do not know [ ]

(B_2_) **Parent’s or Guardian’s Occupation: (**if civil servant please state RANK)

Father ________________________

Mother _______________________

(B_3_) **Family type:** Monogamous [ ] Polygamous [ ]

(B_4_) **Family setting:** Married [ ] Divorced [ ] single [ ] Widowed [ ]

(B_5_) **Does child’s biological father have High blood pressure?** Yes [ ] No [ ] don’t know [ ]

(B_6)_ **Does child’s biological mother have high blood pressure?** Yes [ ] No [ ] don’t know [ ]

(B_7_) **Is your child taking any medication continuously within last 3months?** Yes [ ] No[ ]

If YES, which medication? _______________________

If YES, reason for the medication? ____________________________

(C) SOCIAL HISTORY OF PUPIL (*to be filled by PUPIL on day of study*)

(C_1_) **Do you smoke cigarette?** Yes [ ] No [ ]

(C_2_) **Do you drink alcohol?** Yes [ ] No [ ]

If YES, when was the last time? __________________________

(D) ANTHROPOMETRY AND MEASURES OF ADIPOSITY (***You don’t need to fill this section. This section will be filled by the researcher. I assure you of complete confidentiality of your results***)

Weight (nearest 0.1Kg) _____________

Height (nearest 0.1cm) _____________

Waist circumference (nearest 0.1cm) _______

1^st^ 2^nd^ 3^rd^ Average

Systolic blood pressure (mmHg) _____ ___ ___ ___

Diastolic blood pressure (mmHg) ____ ___ ___ ___

**Derived measures:** BMI ________ Waist to height ratio (WHtR) ____

**Percentiles:** Waist circumference (WC) ___ BMI ____ Systolic BP ___ Diastolic BP
